# A Very High Infection Intensity of *Schistosoma mansoni* in a Ugandan Lake Victoria Fishing Community Is Required for Association with Highly Prevalent Organ Related Morbidity

**DOI:** 10.1371/journal.pntd.0002268

**Published:** 2013-07-25

**Authors:** Edridah M. Tukahebwa, Pascal Magnussen, Henry Madsen, Narcis B. Kabatereine, Fred Nuwaha, Shona Wilson, Birgitte J. Vennervald

**Affiliations:** 1 Vector Control Division, Ministry of Health, Kampala, Uganda; 2 DBL- Centre for Health Research and Development, Faculty of Health and Medical Sciences, University of Copenhagen, Copenhagen, Denmark; 3 School of Public Health, Makerere University, Kampala, Uganda; 4 Department of Pathology, University of Cambridge, Cambridge, United Kingdom; London School of Hygiene and Tropical Medicine, United Kingdom

## Abstract

**Background:**

In schistosomiasis control programmes using mass chemotherapy, epidemiological and morbidity aspects of the disease need to be studied so as to monitor the impact of treatment, and make recommendations accordingly. These aspects were examined in the community of Musoli village along Lake Victoria in Mayuge district, highly endemic for *Schistosoma mansoni* infection.

**Methodology and Principal Findings:**

A cross sectional descriptive study was undertaken in a randomly selected sample of 217 females and 229 males, with a mean age of 26 years (SD ±16, range 7–76 years). The prevalence of *S. mansoni* was 88.6% (95% CI: 85.6–91.5). The geometric mean intensity (GMI) of *S. mansoni* was 236.2 (95% CI: 198.5–460.9) eggs per gram (epg) faeces. Males had significantly higher GMI (370.2 epg) than females (132.6 epg) and age was also significantly associated with intensity of infection. Levels of water contact activities significantly influenced intensity of infection and the highest intensity of infection was found among people involved in fishing. However, organomegaly was not significantly associated with *S. mansoni* except for very heavy infection (>2000 epg). Liver image patterns C and D indicative of fibrosis were found in only 2.2% and 0.2%, respectively. *S. mansoni* intensity of infection was associated with portal vein dilation and abnormal spleen length. Anaemia was observed in 36.4% of the participants but it was not associated with *S. mansoni* infection intensity. Considering growth in children as one of the morbidity indicators of schistosomiasis, intensity of *S. mansoni* was significantly associated with stunting.

**Conclusion:**

Although organ-related morbidity, with the exception of periportal fibrosis, and *S. mansoni* infections were highly prevalent, the two were only associated for individuals with very high infection intensities. These results contrast starkly with reports from Ugandan Lake Albert fishing communities in which periportal fibrosis is more prevalent.

## Author Summary

*Schistosoma mansoni* infection is one of the Neglected Tropical Diseases (NTDs) that perpetuate poverty, especially in Sub Saharan Africa. It is associated with hepatomegaly, splenomegaly or hepatosplenomegaly, liver fibrosis and anaemia. Control of schistosomiasis is now a priority in most endemic countries in Africa as a component of integrated control of NTDs using mass drug administration (MDA). Other than the new WHO strategic plan to eliminate schistosomiasis as a public health problem in WHO Africa region by 2020, the major target in the control of schistosomiasis has for a long time been reduction of its related morbidity. Epidemiological and morbidity studies are key in monitoring the impact of an intervention. However, epidemiology of schistosomiasis and its related morbidity have been shown to vary in different endemic areas and communities. We report on the epidemiology of *S. mansoni* infection and related morbidity in a community in Mayuge District along Lake Victoria in Uganda.

## Introduction

In Uganda, schistosomiasis is mainly caused by *Schistosoma mansoni* [1] and affects more than 10% of the population [2], [3]. It is one of the Neglected Tropical Diseases (NTDs) which perpetuate poverty. The distribution of schistosomiasis has increased due to environmental changes, water development projects and migration of people from endemic to non-endemic areas, spreading to urban areas in developing countries [4], [5].

Schistosome eggs trapped in the host tissue are the major cause of morbidity. Eggs trapped in the liver cause granulomatous reactions and lead to formation of fibrotic lesions with hepatosplenic enlargement which may in turn cause portal hypertension and hepatosplenic schistosomiasis [6], [1], [7], [8]–[10]. Hepatosplenic schistosomiasis is common in children and adolescents and may affect up to 80% of the infected individuals [6], [9] and in the Kenyan study it was exacerbated by malaria otherwise, its severity is related to the intensity of infection [11] and duration of exposure to contaminated water [8]. However, not all infected individuals experience morbidity and the level of schistosomiasis related morbidity differs among affected communities and endemic areas. Other manifestations of schistosomiasis include anaemia [12] and physical retardation [13], [14]. However, other parasitic infections may interact on schistosomiasis-related morbidity. For instance, malaria may act synergistically with schistosomiasis in the development of hepatosplenomegaly [15]–[17] and anaemia is associated with malaria [18] and hookworm [19] infections.

Previously, epidemiological and morbidity data have been reported from the Lake Albert region [1], [11], however such data from Lake Victoria in Uganda are few [20]. This study was carried out to describe the epidemiology of *S. mansoni* infection and its related morbidity among communities living along Lake Victoria.

## Materials and Methods

### Study Area and Population

The study was conducted in Musoli village along Lake Victoria, Mayuge district in South East Uganda. The district lies at an altitude of 1161 m above sea level, with temperatures ranging from 19–27°C and receives annual rainfall in the range of 600–1100 mm [21]. Like other lakes in Uganda, transmission of schistosomiasis in Lake Victoria is stable and intense throughout the year. Musoli village is inhabited by two ethnic groups; the Bantu and Nilotics. The level of literacy is high as compared to other fishing communities in Uganda and most children go to school. Other than subsistence farming, fishing is the major economic activity. Lake Victoria is the only source of water and this exposes the population to schistosomiasis infection.

### Study Design

This was a baseline cross sectional descriptive part of a longitudinal study of randomly selected sample of people stratified by age and sex. Power calculations for multivariate analysis indicated that a minimum sample size of 156 individuals were required. In order to cater for loss to follow up, a higher sample size was selected.

Children 6 years or below were excluded. The study included anthropometric measurements, clinical and ultrasound examinations, haemoglobin assessment and parasitological examinations for *S. mansoni*, hookworms and malaria.

### Parasitological Examination

On three consecutive days, early morning stool specimens were collected from each participant. From each specimen; two slides each containing 50 mg were prepared using the modified Kato Katz thick smear technique [22]. Two experienced technicians examined the six slides under the microscope (10×) within one hour of slide preparation so as to assess presence of hookworm eggs and 24 hours later slides were examined for *S. mansoni*. Hookworm infection was only reported as positive or negative. Intensity of *S. mansoni* infection, described as eggs per gram of stool (epg) was categorised as low: 1–99 epg; moderate: 100–399 epg and heavy: ≥400 epg [23].

A finger prick blood sample was taken from each study subject and a thick blood smear prepared and stained with Giemsa for diagnosis of malaria parasites. The slides were read on a microscope under an oil-immersion objective 40×. A blood slide was considered negative if 100 fields were read and no malaria parasites seen. Malaria parasites were counted against 200 white blood cells (wbc). Assuming a wbc count of 8000/µL the parasite count was multiplied by 40 (8000/200) to calculate number of parasites/µL. Another drop of blood was absorbed into a micro-cuvette, inserted into a portable photometer (HemoCue Hb 201^+^ Analyser,Quest Diagnostics Company, Norrköping - Sweden). Anaemia was defined as: Hb<11.5 g/dL for children 5–11 years; Hb<12.0 g/dL for children 12–14 years; Hb<12.0 g/dL for non-pregnant women ≥15 years; Hb<11.0 g/dL for pregnant women; Hb<13.0 g/dL for men ≥15 years, [24]. As quality control, two independent technicians read a 10% randomly selected sample of stool and malaria slides respectively.

### Individual Interviews

A questionnaire was developed and translated into the most commonly used language, Lusoga. The questionnaire was used to obtain information about individual water contact exposure patterns and ethnic group. For children who could not answer some of the questions, their parents or guardians provided the required information.

### Anthropometric Measurements

Height was measured to the nearest 0.1 centimetres using a portable stadiometer and weight measured to the nearest 0.1 kg using a Seca portable digital scale. Z scores of height-for-age (HAZ) and weight-for-age (WAZ) were calculated using Nutritional Index Calculator, EpiInfo, Version 6.04 (Centers for Disease Control and Prevention, USA). HAZ and WAZ values less than -2 were considered as stunting and wasting respectively as described by WHO [25]. Body Mass Index (BMI) of each child was calculated as weight in kilograms divided by the square of height in meters (kg/m^2^). In our study, BMI<15 kg/m^2^ was considered as underweight, otherwise BMI beyond 15 kg/m^2^ would have rendered all the children in our study to be underweight.

### Clinical Examination

Each individual was clinically and independently examined by three experienced examiners, i.e. one physician and two nurses. The obtained measurements from all the examiners were discussed and a final measurement agreed upon. If the measurements of the three examiners varied greatly, all the examiners repeated the examination.

Abdominal palpations were performed as previously described [12]. Using a tape measure, the following measurements were taken: the extension of the left liver lobe beneath the sternum was measured in the mid sternal line (MSL); the extension of the right liver lobe beneath the rib cage was measured in the right mid clavicular line (MCL); the extension of the spleen below the rib cage was measured both in the left MCL and left mid axillary line (MAL). The liver tenderness and its consistency as well as the spleen consistency were graded as described by Vennervald [12]. The findings were translated into an overall clinical score reflecting the degree of organomegaly. The consistency of the organs and any signs of portal hypertension were recorded.

### Ultrasound Examination

Ultrasonography was performed by two experienced ultrasonographers using a portable ultrasound machine (SSD 500 Aloka with 3.5 MHz curvilinear - 60% probe). Each individual was examined by one ultrasonographer, who would consult another examiner in case of need. The subjects were examined in a supine position lying with their legs stretched on an examination coach. The portal vein diameter was measured at the porta hepatis at the ventral lower end of the caudate lobe as previously described [26]. Liver texture patterns were graded according to WHO guidelines [27]. Portal vein diameter values were compared with the values for the corresponding height groups from a Senegalese non-infected population and classified as normal if they were below or equal to the mean+2 SD; moderately abnormal if they were >2 SD but ≤mean+4 SD and severely abnormal if they were >mean+4 SD [28] as suggested by Richter [27].

### Statistical Procedures and Data Analysis

The data were double entered in Microsoft Excel and analysis performed using Stata 11.0 (Stata Corporation, USA). *Schistosoma mansoni* egg counts and malaria parasite counts were analysed in relation to various predictors using negative binomial regression [29] adjusting for clustering within households; we used generalized linear models using log-link function. The ancillary parameter was estimated using full maximum likelihood estimation [29] and was used in the generalized linear model. For this analysis *S. mansoni* egg counts were summed for all slides (up to 6; less than 8% did not provide the full set of samples) and total faeces examined ( = no. of slides*0.05 g) was entered as an offset. Similarly, prevalence of infection by *S. mansoni*, malaria or hook worm infections and of various morbidity indicators (anemia, portal vein dilatation, organomegaly) was analysed using logistic regression [30] adjusting for possible clustering within households. The model building strategy was to test all potential predictors one by one after adjusting for sex and age group whether these were significant or not and the interaction between these two factors if significant. Significant predictors were then entered together with sex, age group and the interaction between the sex and age group. Insignificant predictors were then eliminated including possibly some of the indicator variables for levels in categorical variables. Model fit for count models was assessed using dispersion statistics to check for over dispersion and Anscombe residuals were used to check for outliers [29]. Logistic regression models were assessed using the Hosmer-Lemeshow goodness of fit statistic [30]. P-values less than 0.05 were considered significant.

### Ethical Consideration

Ethical approval was obtained from the Higher Degrees Research and Ethics Committee of the School of Public Health, Makerere University. Ethical clearance was granted by the Uganda National Council of Science and Technology and the Danish National Committee on Biomedical Research Ethics in Denmark. Written in the local language, consent forms were used to obtain individual adult participants' consent while parents or guardians consented on behalf of participants less than 15 years. Participants suffering any minor ailment like clinical malaria, anaemia, diarrhoea and others were treated according to the Uganda national guidelines. Following the National Schistosomiasis Control Programme guidelines, all study participants were treated with 40 mg/kg body weight of praziquantel (Distocide 600 mg) and one tablet of albendazole (Alzental 400 mg) all manufactured by Shin Poong Pharmaceuticals, Seoul Republic of Korea, irrespective of their infection status. The rest of the community were treated following the National guidelines.

## Results

A total of 446 people, 229 males and 217 females, with a mean age of 25.8 years (range 7–68 years) for males and 25.2 years (range 7–76 years) for females, were examined. There were 161 (36.1%) farmers, 70 (15.7%) fishermen, 177 (39.7%) students, while 38 (8.5%) were of other occupations. With regard to ethnicity, 241 (54.0%) were Bantu of the following major tribes: Baganda, Basoga, Basamia and Bagwere. The other 205 (46.0%) were Nilotics (Japadhola, Acholi, Alur and Iteso tribes). The water contact activities reported in this study were washing clothes or utensils, fetching water, swimming, bathing and fishing. Fishing was reported as the only activity by 4.0%, fetching water as only activity by 26.3%, swimming, washing clothes and fetching water by 53.3% and all (fishing, swimming, washing clothes and fetching water) activities were reported by 16.3%. Considering the frequency of exposure, 73.4% of the people reported to go to the lake more than 5 days in a week while 13.0% and 13.6% went there 3–4 days and 1–2 days in a week respectively.

### *S. mansoni* Infection Levels

**Figure 1:**
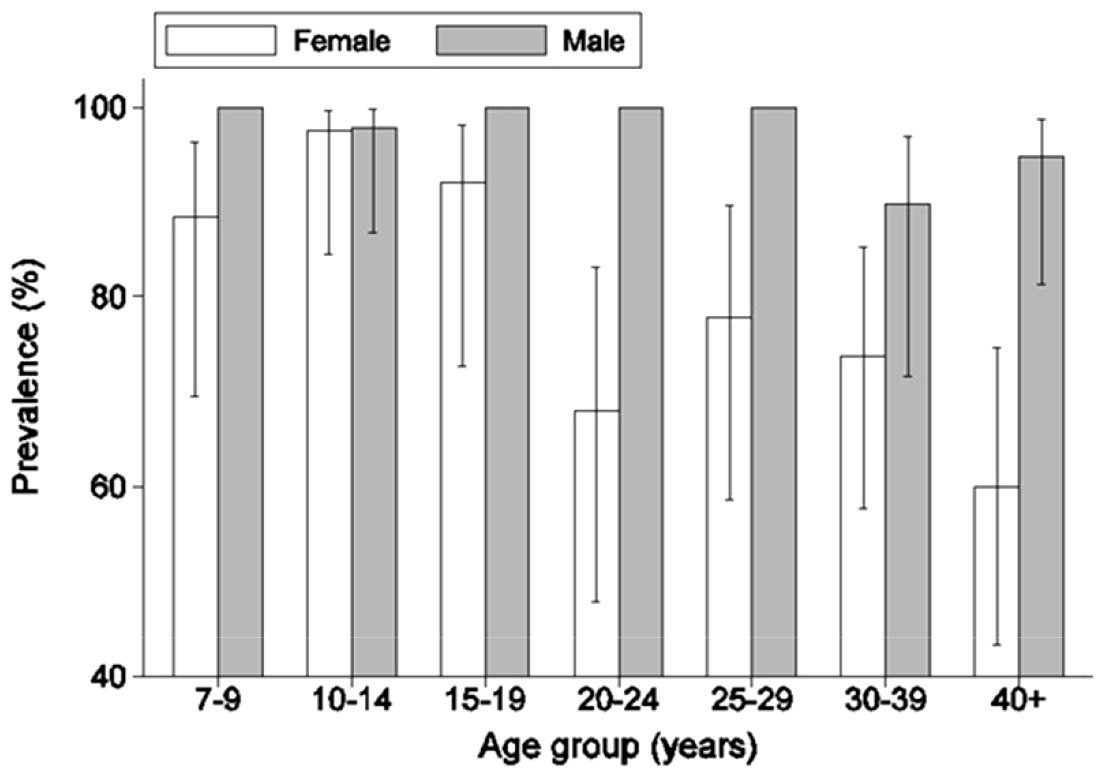
Prevalence of *S. mansoni* infection by sex and age group. Error bars show 95% confidence limits based on the logistic regression model.

The overall prevalence of *S. mansoni* was 88.6%. The overall GMI for positives only was 236.3 epg (95% CI: 198.5–460.9) and overall proportions of heavily, moderately and lightly infected persons were 39.0%, 22.2% and 27.4% respectively. Males generally had higher prevalence of infection than females in all age groups, and the interaction between sex and age group was significant (p<0.001). Basically, all males in the age range 7 to 29 years were infected and prevalence of infection among females was high in the age range from 7 to 19 years while prevalence among females aged 20 years and above was low (Fig. 1). The following factors tested individually and after adjusting for age, sex, the interaction between sex and age group and clustering within households were significant, ethnic group (p<0.001), occupation (p<0.05) and water contact activity (p<0.001). The significance of ethnic group was mainly due to the Bagwere all being infected (n = 27). For occupation, students were reported to have higher odds of infection while for water contact people reporting to be involved in fishing had higher odds of infection. Duration of stay was coded as 1–2 years, 3–4 years, 5–9 years, 10–14 years and 15 or more years. Odds of infection among those with a duration of stay of 1–2 years was 5.2% (p<0.001) and those with a duration of 3–4 years was 11.9% (p<0.001), of those of people with a duration of stay of 15 or more years.

The final model showed that gender (<0.001), age group(<0.01), the interaction between gender and age group (<0.001), being a student (OR = 109.7; p<0.01), having stayed in the area 1–2 years (OR = 0.07 p<0.001) or 3–4 years (OR = 0.2 p<0.01) compared to people having stayed 5 or more years; and engagement in fishing (OR>1000; p<0.001) were all significant. The sample size was reduced to 381, since some people did not report on water contacts (frequencies and type of activity).

**Figure 2:**
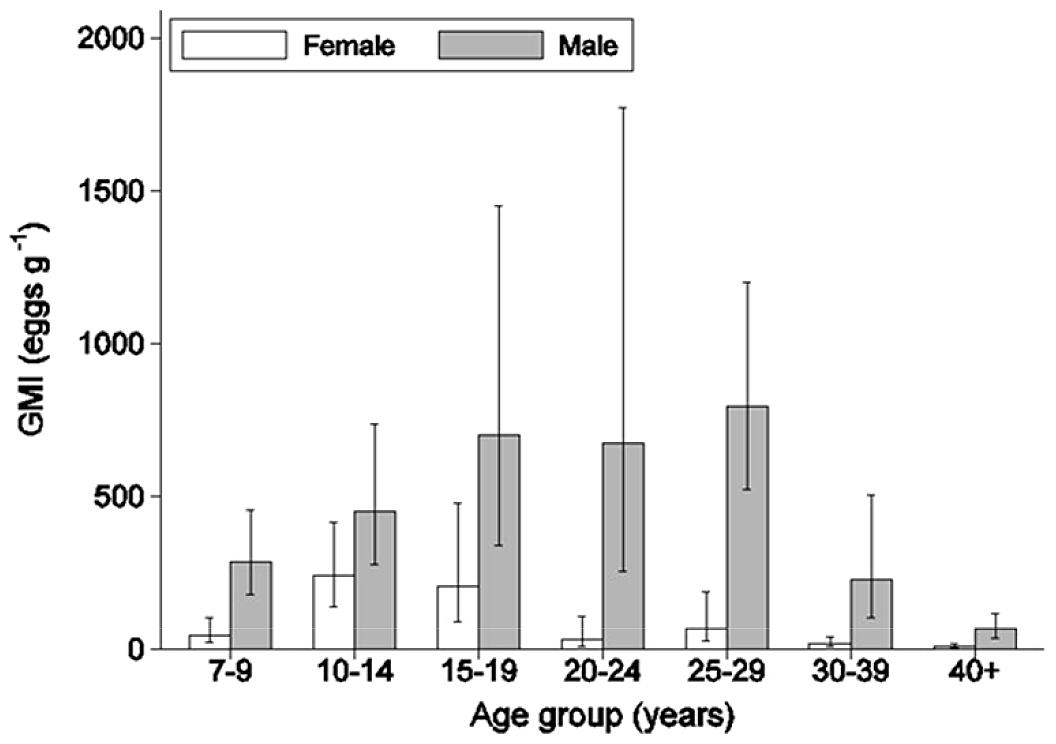
Geometric mean intensity (GMI) of *S. mansoni* infection (eggs g^−1^) by sex and age group. Error bars show 95% confidence limits.

Intensity of infection varied greatly among individuals with a maximum egg count of 7083 epg. Males had on average 3.40 times higher egg counts than females (Fig. 2; Table 1) and intensity of infection varied significantly across age classes (p<0.001), while the interaction between sex and age groups was not significant.

**Table 1:** *S. mansoni* infection intensity by sex and age.

Characteristic	No. +ve	*GMI	95% CI
Overall	395	236.2	198.5–460.9
**Sex**			
Female	173	132.6	102.5–171.5
Male	222	370.2	297.5–460.9
**Age group by sex (years)**			
**Female**			
7–9	23	76.5	40.5–144.6
10–14	40	275.3	169.9–446.2
15–19	23	327.9	187.2–1144.6
20–24	17	170.2	72.8–398.0
25–29	21	223.5	120.4–414.8
30–39	28	54.9	27.3–110.4
40+	21	35.1	17.1–72.3
**Male**			
7–9	39	284.6	178.0–455.0
10–14	46	513.7	336.6–783.8
15–19	21	700.7	338.7–1449.6
20–24	16	671.9	254.4–1774.3
25–29	29	793.1	523.0–1202.5
30–39	35	421.3	234.0–758.6
40+	36	81.9	49.2–136.3

The following factors tested individually after adjusting for age, sex, and clustering within households were significant: occupation (p<0.01; fishing being associated with higher intensities of infection), water contact activity (p<0.001; those reporting fetching of water only had lower egg counts than those involved in all reported activities), frequency of visiting the lake (p<0.05; with 5–7 times per day being associated with higher intensity of infection), and duration of stay (<0.01). Egg counts as percentage of egg counts among people having stayed 15 or more years were 38.5% (p<0.01), 55.5% (n.s.) and 61.4% for people having stayed 1–2 years, 3–4 years and 5–9 years respectively after adjusting for gender and age group and clustering within households. The final model showed that males had higher egg counts than females, and lower egg counts were associated with people who reported to have water contact 1–2 days per week, those reporting to only fetch water and those having stated only for 1–2 years in the area (Table 2).

**Table 2:** *S. mansoni* infection intensity by water contact and duration of stay in the village.

Characteristic	No. +ve	*GMI	95% CI
**Frequency of water contact (days/week)**			
1–2	35	110.5	59.4–205.7
3–4	37	249.7	142.2–438.4
5–7	213	276.6	219.3–348.9
**Water contact activity**			
Fishing	16	590.7	226.7–1538.8
Fetch water	80	108.2	74.5–157.0
Swim/wash/fetch water	182	195.6	151.1–253.2
All activities	59	792.4	581.0–1080.8
**Length of stay (years)**			
1–2	34	176.3	96.6–321.6
3–4	25	240.4	115.1–502.0
5+	336	242.9	201.2–293.4

### Infection with Malaria and Hookworm

The overall prevalence of hookworm infection was 43.3% and did not differ significantly between sexes. Prevalence of hookworm infection increased with age group up to the age group 20–24 years while differences between this age group and those older were not significant. Odds ratios for the age groups 7–9 years, 10–14 years and 15–19 years compared to all older age groups combined were 0.25 (p<0.001), 0.21 (p<0.001) and 0.49 (p<0.05), respectively. Prevalence of infection was not related to the following factors tested individually after adjusting for age effects and clustering within villages: ethnic group, occupation, frequency of water contact, water contact activities or malaria prevalence.

Malaria parasitaemia was found in 291 (65.2%) of the participants with a mean parasite density of 571.4 parasites/µL (95% CI: 430.8–712.1). Prevalence of malaria parasitemia declined with increasing age (p<0.001) from age group 7–9 years to the age group 20–24 years, while differences between the latter and the older age groups were not significant. Odds ratios for the age groups 7–9 years, 10–14 years and 15–19 years compared to all older age groups combined were 9.91 (p<0.001), 5.82 (p<0.001) and 2.86 (p<0.05), respectively. Prevalence of malaria parasitemia was not related to the following factors tested individually after adjusting for age effects and clustering within households: ethnic group, frequency of water contact, water contact activities or malaria prevalence, while occupation was significant (p<0.05) with students having slightly higher levels of infection. A total of 246 people (59.4%) were co-infected with *S. mansoni* and malaria while 106 (23.8%) had *S. mansoni*, hookworm and malaria.

Malaria parasite density did not differ significantly between sexes when after adjusting for age group, while density decreased with increasing age from 5–9 years to the 15–19 years age group (p<0.001). Density did not differ significantly between the 15–19 years age group and those older. Parasite count ratios for the age groups 7–9 years and 10–14 years compared to all older age groups combined were 6.20 (p<0.001) and 3.70 (p<0.001), respectively. Parasite counts in the 10–14 years age group was 60% of those in the 7–9 years age group, while in the 15–19 years age group it was 24% of that in the 7–9 years age group. Malaria parasite density of positives only was 884.8 parasites/µL (95% CI: 600.3–1169.3) in children aged 7–14 years and 303.9 parasites/µL in adults 15 years or older (95% CI: 226.4–381.5).

### Organomegaly

From clinical examinations, after analysing each organomegaly indicators separately, levels of hepatomegaly, splenomegaly and hepatosplenomegaly were moderate. Overall presence of hepatomegaly was 24.2%, splenomegaly 4.9% and hepatosplenomegaly 30.3%.

Hepatomegaly did not differ between sexes after adjusting for age groups, while variation across age groups was significant (p<0.05) with adults having slightly lower prevalence of hepatomegaly. None of the following factors were significant when tested individually after adjusting for age category: ethnicity, occupation, water contact activities, *S. mansoni* intensity category, malaria or hookworm infection. *S. mansoni* intensity category tested as the only predictor after adjusting within households was not significant. Length of stay was not associated with hepatomegaly when adjusting for age group.

Splenomegaly did not differ between sexes or among age groups and none of the factors listed above for hepatomegaly were significant. Length of stay was not associated with splenomegaly when adjusting for age group.

Hepatosplenomegaly did not differ among sexes when adjusting for age group, while it varied among age groups (p<0.01) with age groups 15–19 years and older having less hepatosplenomegaly than the 7–9 year class. The odds ratio for adults (15 years and above) compared to children was 0.44. Among other factors tested individually after adjusting for age group and clustering within households only malaria infection was associated (p<0.05) with increased hepatosplenomegaly (OR = 1.75, 1.04–2.94).

The odds of having hepatosplenomegaly among the very heavily infected (n = 41) was 3.39 times higher than among people with no infection or with less than 2000 epg (p<0.01) when adjusting for age class. This category of very heavy infections, however, was not associated with splenomegaly or hepatomegaly. Of those infected with *S. mansoni* and having enlarged spleens and livers, majority had firm organs (92.7% and 89.9% respectively). Length of stay was not associated with hepatosplenomegaly.

Periportal fibrosis (PPF) was rare. A total of 423 (94.8%) and 3 (0.7%) had normal livers with patterns A and B respectively, while ten (2.2%) and one (0.2%) people had liver image patterns C and D respectively. Patterns Z and Y, associated to alcoholic-related liver damage, were observed in 1 (0.2%) and 8 (1.8%) people respectively.

**Figure 3:**
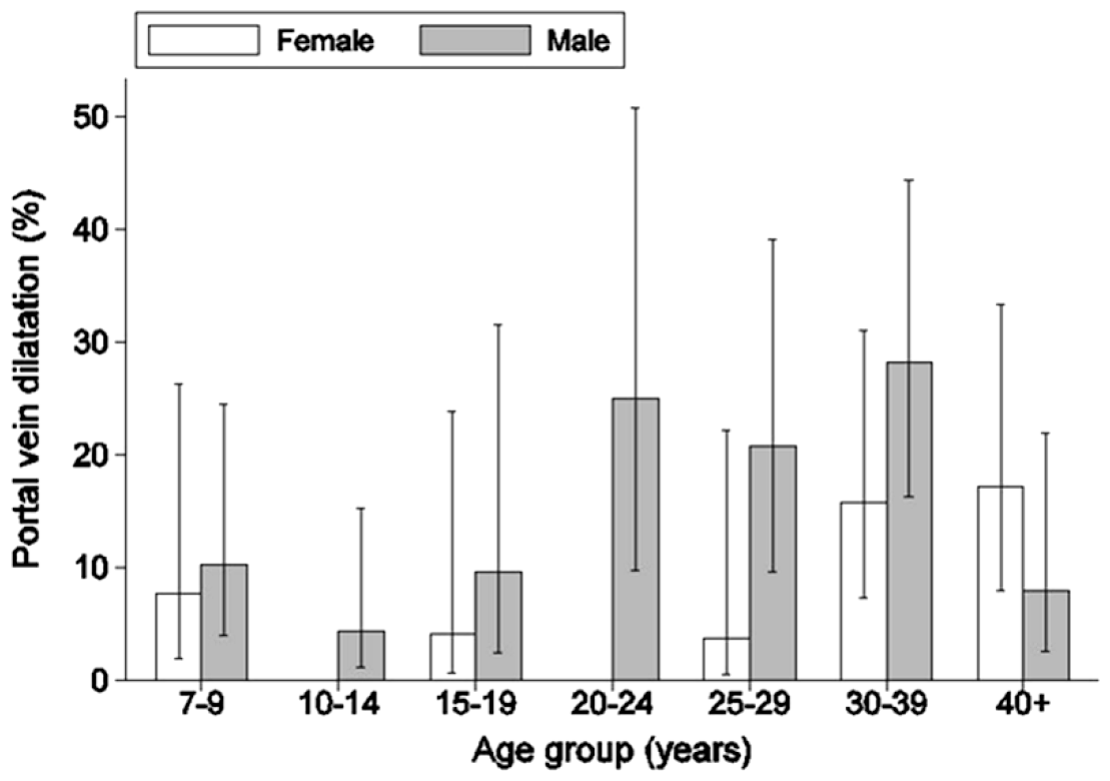
Prevalence of portal vein dilatation by sex and age group. Error bars show 95% confidence limits based on the logistic regression model.

A total of 48 people (10.8%) had dilated portal vein diameters. Portal vein dilatation differed significantly between sexes (p<0.05) and among age groups (p<0.05). There was, however, a significant interaction between sex and age group (Fig. 3). Apart from the youngest age group few females had dilated portal veins in the age groups up to the 25–29 years class, while in the older classes females had higher prevalence of dilated portal vein. Among the other factors tested individually after adjusting for sex, age group, the interaction between these two factors and possible clustering within households, occupation (p<0.05; with fishers having the highest prevalence) and water contact activities (p<0.01; with those involved in all forms of activity having highest prevalence) were significant. Intensity categories for *S. mansoni* infection were not related to portal vein dilatation. However, the odds of having dilated portal vein among the very heavily infected (n = 41) was 4.49 (p<0.001) times higher than among people with no infection or with less than 2000 epg after adjusting for age. Length of stay in the villages was not associated with dilated portal vein prevalence after adjusting for age and clustering within households.

### Effect of *S. mansoni* on Haemoglobin Levels and Growth

Haemoglobin (Hb) level, (overall value 12.6 g/dL, 95% CI: 12.4–12.8) differed between sexes and across age groups, but there was a significant interaction these two variables (p<0.001). None of the other factors evaluated individually after adjusting for sex, age group, the interaction between these two factors and possible clustering within villages were significantly associated with haemoglobin levels, i.e. ethnic group, occupation, water contact, *S. mansoni* intensity category, malaria infection and hookworm infection. Overall 36.4% (95% CI: 31.7–41.1) of the people were anaemic but it was not associated with sex, age group, intensity of infection by *S. mansoni*, malaria nor hook worm infection.

Intensity of *S. mansoni* significantly affected the level of stunting (p<0.01). However, there was no evidence of the effect of schistosomiasis on wasting and underweight (Table 3).

**Table 3:** *S. mansoni* infection intensity by growth indicators.

Indicator	GMI (N)	95% CI
**HAZ**		
Normal	227.4 (120)	170.8–302.8
Stunted	571.3 (29)	330.3–988.2
**WAZ**		
Normal	262.3 (139)	201.3–341.8
Wasted	463.1 (12)	160.5–1336.8
**BMI**		
Normal	274.3 (129)	209.0–359.9
Underweight	275.2 (22)	124.6–608.0

## Discussion

Schistosomiasis is highly prevalent (88.6%) with high intensity of infection in this Victoria Lake shore community and infection is related to water contact. The high infection level is typical for endemic areas around Lake Victoria [7], [31]–[33]. Intensity of infection is higher in males than females [2], [10], [34]. This could be due to occupational exposure such as fishing [35], which prolongs the duration of contact with schistosome-infested water [8], [36].

The peak *S. mansoni* infection intensity occurred in the 15–19 year age group, similar to observations elsewhere [2], [10], [37]–[39]. Several explanations have been suggested for this trend, among which is water contact. However, exposure alone may not explain this age difference in infection. A study of a fishing community along Lake Albert, where adults were more exposed to infested water than children recorded a similar age infection pattern [10]. This pattern could be explained by slow development of acquired immunity to schistosomiasis infection. In endemic areas, people acquire immunity in response to parasite antigens and this immunity is influenced by age [40], [41] or duration of exposure [42], [43]. Another explanation for infection peaking in the second decade of life could be due to physiological changes at puberty [38]. Hormonal changes during puberty, such as increase in skin thickness or deposition of fat, increase resistance to *S. mansoni* infection by reducing cercarial penetration [42], [44].

The most common observed morbidity indicator in our study, hepatosplenomegaly without fibrosis, concurs with observations from a Kenyan study [19]. Having more children than adults with hepatosplenomegaly, is also comparable to other studies [28], [45]. Could the age difference in prevalence of hepatosplenomegalybe due to increased regulation of inflammatory immune responses with age [41], [43]? Contrary to other studies where hepatosplenomegaly was associated with prevalence of *S. mansoni* [46], [47] and intensity of infection [9], [13], [48], clinically detected enlarged spleens and livers in our study were not associated with prevalence of *S. mansoni*. Our findings are comparable to those among Kenyan school children where no significant difference in hepatosplenomegaly between *S. mansoni* infected and un-infected children was found [17]. The observed organomegaly is likely to have been affected by the highly prevalent malaria infection in our study as evidenced by the weak correlation we obtained between the liver size and malaria infection. However, when heavy intensity of *S. mansoni* infection was further categorised, hepatosplenomegaly showed an association with very heavy intensity. This is in agreement with earlier studies in Uganda [11], [49] and else where [50].

Our study was carried out in a community with more or less similar living conditions as those in a study conducted along Lake Victoria in Tanzania [13] that had lower GMI than what we obtained and another one along Lake Albert [11] but contrary to our study, these two studies registered high levels of PPF. The low PPF levels in our study were not expected because markedly high morbidity has been reported by other studies along Lake Albert [8], [11] and also from evaluation of the impact of the Uganda National NTD Control Programme in areas where *S. mansoni* infection intensity was the same as what we obtained in our study [51].

In one of the studies along Lake Albert, a lower prevalence of PPF was attributed to shorter duration of exposure to infection [8]. In our study, duration of exposure is not likely to have affected PPF since most people were born in the village and having no other source of water; all of them are exposed to infection. Probably factors like parasite genetic differences [28] could have influenced the levels of PPF we obtained. This is supported by findings from a study that revealed a genetic variation of *S. mansoni* parasites in Lake Albert from those of Lake Victoria [52], more so with locally different strains within Lake Victoria [53].

Anaemia levels were mild and comparable with findings from other studies [19], [54]. In a Kenyan study, no relationship between Hb and intensity of *S. mansoni* was observed and this was attributed to low intensity of *S. mansoni* infection [19]. Although we realised high *S. mansoni* intensity of infection than the Kenyan study, levels of infection had no effect on anaemia. It should be noted that schistosomiasis may not be the only predictor but other parasitic infections such as malaria and hookworms may cause anaemia [12], [18], [54]. Nonetheless, anaemia was neither associated with malaria nor hookworm infections in our study. This is also contrary to previous studies which report an association between malaria and anaemia [18], [19] yet malaria parasitaemia was as prevalent in our study as it was in these studies. Other factors such as poor nutritional diet or inadequate dietary in-take without iron supplements, poverty and haemoglobinopathies may also have an impact on haemoglobin levels and lead to anaemia [18], [55]. Our results suggest that anaemia was probably a result of other factors such as poor nutrition, haemoglobinopathies, increased haemolysis in the spleen or hookworm intensity of infection [56], all of which were not assessed in our study.

Growth parameters were computed for only children below 15 years of age because this age group is most susceptible to debilitating effects of schistosomiasis infection [14], [57]. Other than stunting, wasting and underweight were not associated with schistosomiasis infection intensity. This is contrary to the Kenyan study where no significant relationship between stunting and *S. mansoni* infection was observed [58].

In conclusion, *S. mansoni* infection is highly prevalent in Mayuge district. Whereas there is evidence from this study that an individual's age, sex and occupation influence the level of *S. mansoni* intensity of infection, only severe heavy intensity of infection has an influence on morbidity. Furthermore, the schistosomiasis specific periportal fibrosis was practically absent. These results indicate that organ related morbidity cannot solely be used as an indicator of successful morbidity reduction when monitoring the impact of a schistosomisis control programme. This is clearly illustrated by this study in combination with previous reports which show that morbidity differs between endemic areas with similar exposure patterns. There is a complex relationship between intensity and organ related morbidity, with these currentresults contrasting starkly with reports from Ugandan Lake Albert fishing communities where periportal fibrosis was more prevalent.

## Supporting Information

Checklist S1 **STROBE Checklist.**
(DOC)
